# Gender Differences in Survival and the Use of Primary Care Prior to Diagnosis of Three Cancers: An Analysis of Routinely Collected UK General Practice Data

**DOI:** 10.1371/journal.pone.0101562

**Published:** 2014-07-11

**Authors:** Yingying Wang, Nick Freemantle, Irwin Nazareth, Kate Hunt

**Affiliations:** 1 MRC|CSO Social & Public Health Science Unit, University of Glasgow, Glasgow, United Kingdom; 2 Department of Primary Care and Population Health, University College London, London, United Kingdom; National Cancer Center, Japan

## Abstract

**Objective:**

To explore whether there are gender differences in the number of GP recorded cases, the probability of survival and consulting pattern prior to diagnosis amongst patients with three non-sex-specific cancers.

**Design:**

Cross sectional study.

**Setting:**

UK primary care.

**Subjects:**

12,189 patients aged 16 years or over diagnosed with colorectal cancer (CRC), 11,081 patients with lung cancer and 4,352 patients with malignant melanoma, with first record of cancer diagnosis during 1997–2006.

**Main outcome measures:**

Cancer cases recorded in primary care; probability of survival following diagnosis; and number of GP contacts within the 24 months preceding diagnosis.

**Results:**

From 1997–2006, overall rates of GP recorded CRC and lung cancer cases recorded were higher in men than in women, but rates of malignant melanoma were higher in women than in men. Gender differences in survival were small; 49% of men and 53% of women survived at least 5 years following CRC diagnosis; 9% of men and 12% of women with lung cancer, and 77% of men and 86% of women with malignant melanoma. The adjusted male to female relative hazard ratio of death in all patients was 1.20 (95%CI 1.13–1.30), 1.24 (95%CI 1.16–1.33) and 1.73 (95%CI 1.51–2.00) for CRC, lung cancer and malignant melanoma respectively. However, gender differences in the relative risk were much smaller amongst those who died during follow-up. For each cancer, there was little evidence of gender difference in the percentage who consulted and the number of GP contacts made within 24 months prior to diagnosis.

**Conclusions:**

This study found that patterns of consulting prior to cancer diagnosis differed little between two genders, providing no support for the hypothesis that gender differences in survival are explained by gender differences in consultation for more serious illness, and suggests the need for a more critical view of gender and consultation.

## Introduction

Women live longer than men in almost all countries [1]. Both biological and social explanations have been posited for this gender difference [2]. Bio-medical research has focused on anatomy, physiology and the role of sex hormones in explaining differences between health outcomes in men and women [3], whilst sociological research has emphasised behavioural and cultural factors. In particular, differences in health behaviours such as smoking, heavy drinking and poor diet appear to explain much of the gender differences in mortality apparent over recent decades [4], [5]. Well documented gender differences in the frequency of contact with primary health care services, in particular in early adult life and middle age, based both on self-reported [6] and routinely collected consultation data [7], [8], have led many to suggest that differences in health service use are also an important contributor to gender differences in mortality. Such arguments are bolstered by qualitative studies which have documented men's apparent reluctance to consult [9], [10]. Despite recent reviews which challenge this view [11], [12], there remains a widespread assumption that men are *always* more reluctant to consult and that, by extension, gender differences in the propensity to consult may partly explain gender differences in longevity (through delayed consultation leading to later diagnosis and therefore decreased opportunities for effective treatment and reduced survival in men). However, there is a paucity of research comparing consultation patterns in men and women with similar underlying (and serious) morbidity to support or refute the claim. Here we present an analysis of consultations made by cancer patients as recorded in a large general practice database, examining consultation pattern amongst men and women in the 24 months prior to a recorded diagnosis with three non-sex specific cancers, namely colorectal cancer, lung cancer and malignant melanoma.

In the UK, as in other parts of Europe, men are at greater risk of being diagnosed with, and dying from, nearly all non-sex specific cancers [13], although gender patterns have changed over time. Between 1975 and 2010, cancer incidence rates in the UK increased by 22% in men and 42% in women but cancer mortality decreased more rapidly in men than women (by 28% and 16% respectively). These trends partly reflect changing patterns of smoking by gender in earlier decades. In 2010, 324,579 new cases of cancers (excluding non-melanoma skin cancer) were diagnosed in the UK, including 163,904(50.5%) in men and 160,675 (49.5%) in women. There were 82,481 male deaths and 74,794 female deaths from cancer in the same year [13], [14]. Of non-sex specific cancers, lung cancer is the most common cause of death for both genders, accounting for 24% of cancer deaths in men and 21% in women, and colorectal cancer (CRC) is the second biggest cause of cancer death (accounting for 11% and 10% of cancer deaths in men and women respectively) [15], [16]. Between the ages of 15 and 64 years, malignant melanoma is one of the few non-sex specific cancers to be more commonly diagnosed in women than men, although the gender pattern is reversed at older ages [13].

If gender differences in *consulting* are important in explaining gender differences in *mortality*, we should expect that men would consult primary health care services less *and* that they would consult later with symptoms that could be indicative of major contributors to overall death rates, such as these common forms of cancer. Analyses of general practice consultation data do indeed provide evidence that women consult more on average than men *overall*, particularly in early adulthood and mid-life [6], [8]. However, the evidence on whether there are gender differences in patterns of consultation prior to diagnosis with colorectal cancer is mixed. An early North American study showed a significantly longer delay from first noting symptoms to diagnosis in *women* rather than men, contradicting widespread assumptions, and a longer delay for women between first consultation for the symptoms and diagnosis (“physician delay”) [17], whilst a recent population-based survey reported no gender differences in seeking help for cancer symptoms such as rectal bleeding [18]. Other studies have noted men's greater likelihood of delaying help-seeking following the development of CRC symptoms. For example, an Australian study of people with CRC found that a higher percentage of men than women (31% and 10% respectively) waited more than 3 months from initial symptoms to their first visit to their doctors [19]. Studies of gender differences in time to consultation for symptoms of lung cancer are also contradictory. An early American study suggested that women experienced a longer delay than men between first recognition of symptoms and lung cancer diagnosis, although the absolute difference between the two was small [20], but a Scottish study found no gender differences in time to consultation [21] and a recent pilot study in the UK found no difference between men and women in terms of symptom times and presentation to the GP [22].

Given this background, this paper presents analyses of routinely collected data from the Health Improvement Network, a large UK primary care database. Our aim is to assess whether there is any evidence to support the hypothesis that gender differences in patterns and timing of consulting for symptoms of non-sex specific cancers (i.e. male ‘reluctance’ to consult until later stages of diseases) could plausibly account for gender differences in mortality. We approach this question in three stages. First, we examine gender-specific rates of three cancers (CRC, lung cancer and malignant melanoma) by age and deprivation levels, to allow comparison of the gender patterns in cancer rates observed in participating general practices within THIN with other national sources. Secondly, since stage of cancer is not reliably recorded in the routine data source, we investigate whether there are gender differences in survival following diagnosis with these three non-sex specific cancers; if men were presenting at a later stage, we would expect that their survival, especially in the first few years after diagnosis would be worse. Thirdly, we query whether there are gender differences in the number of GP contacts within the 24 months preceding diagnosis. Our hypothesis is that, if gender differences in the use of health services are an important contributor to gender differences in mortality, we would expect poorer survival following diagnosis in men, particularly in the years soonest after diagnosis, and marked gender differences in the number and time of patients' contacts with their GPs prior to diagnosis.

## Methods

### Data source

UK general practices are usually the first point of contact for UK patients using the National Health Service (NHS). The Health Improvement Network (THIN) database is one of the largest primary care data sources, consisting of electronic records for over 11 million patients from more than 500 general practices in the UK. THIN contains anonymised patient data directly extracted from practices using the Vision general practice system and is a clinical database which includes information on patients' year of birth, gender, post code, registration details, clinical symptoms, medical diagnoses, laboratory tests, referrals and prescriptions, at every primary health care service contact. Participating practices are broadly representative of UK general practice [23], [24]. The database also includes information on individual patients' socio-economic status (measured by quintiles of Townsend deprivation score based on 2001 census data). Information about deaths is recorded in THIN in a specific field in an additional health file; for this analysis an individual was accepted as dead if either there was a record of date and cause of death or if a death certificate or other external document confirmed the death.

### Study population

In UK primary care, diagnoses are recorded using Read codes, a hierarchical classification system that includes terms relating to signs and symptoms, diagnosis, procedures and investigations [25]. Clinical diagnoses made by specialists and results from diagnostic tests are entered retrospectively in general practice. Previous studies have shown that the recording of cancer cases in THIN is representative of cancer cases captured in other national statistics [26], [27]. For the current study, we identified all permanently registered patients (aged 16 years or over)in THIN with a first recorded diagnosis of lung cancer, colorectal cancer, or malignant melanoma between 1997 and 2006 to allow us to examine survival for a minimum of five years in all cancer cases as the availability of latest data was to 31.12.2011, although follow up for earlier cases is potentially much longer (up to 15 years). Read codes used to identify cases of a diagnosis of each of the three cancers are available on request.

### Statistical analysis

Rates of each of the three cancers were calculated by dividing the number of cancer cases recorded by the number of person years at risk. The denominator included all patients who had contributed data after 1^st^ January 1997, and rates were calculated for the study period between 1997 and 2006. Gender-specific rates are presented by age groups and quintiles of Townsend scores.

In the survival analysis, we used a Kaplan-Meier estimator to compare men's and women's probability of survival over the 5 years following cancer diagnosis, and log-rank tests were used to estimate survival difference in men and women. Since stage of cancer is not reliably recorded in the THIN database, we examined five year survival first in *all* patients with each cancer, and then in the subset who had died at some time during follow-up (minimum follow-up is five years, maximum follow-up is 15 years), as a proxy for severity. Poisson Mixed Regression models are applied to estimate gender differences in relative hazard of death when age and socio-economic deprivation status were adjusted for, and fitted survival models with time varying random effects (defined by subject) to account for non linearity in relative hazards. This analysis was applied to all cancer patients diagnosed between 1997 and 2006 and to the subset who died (all cause death) at some time following cancer diagnosis.

We then identified all primary care contacts between clinicians and patients diagnosed with CRC, lung cancer and malignant melanoma between 1997 and 2006 within the 24 months preceding their diagnosis. Consultation rates were calculated using number of consultations recorded prior to diagnosis as the numerator and the number of cancer patients as the denominator. We compared consultation rates in men and women. A Poisson regression model was used to estimate gender difference in consulting rate prior to cancer diagnosis.

Analyses were conducted in Stata 12.

## Results

### 1. Rates of cancer by gender

In total, between 1997 and 2006, 12,189 patients aged 16 years or over were diagnosed with CRC, 11,081 patients with lung cancer and 4,352 patients with malignant melanoma.

For colorectal cancer, 6,532 cases (54%) were diagnosed in men (median age 71 years, interquartile range (IQR) 63–78) and 5,657(46%) in women (median age 74 years (IQR = 64–81)) (Table 1). The overall rate of CRC cases recorded during the study period was 68.30 (95% CI 66.66–69.85) per 100,000 person years in men and 56.86 (95%CI 55.39–58.37) per 100,000 person years in women. Rates of CRC cases rose with increasing age in both men and women, and there was little gender difference in the rates of CRC cases recorded before the age of 50, after which gender differences increased. CRC rates were consistently higher in men than women across all quintiles of Townsend scores.

**Table 1:** Number of cancer cases stratified by gender, age groups and deprivation quintiles (1997–2006).

	Colorectal Cancer				Lung Cancer				Malignant melanoma			
	M		F		M		F		M		F	
	No. of cases	No. of cases/100,000 PYAR	No. of cases	No. of cases/100,000 PYAR	No. of cases	No. of cases/100,000 PYAR	No. of cases	No. of cases/100,000 PYAR	No. of cases	No. of cases/100,000 PYAR	No. of cases	No. of cases/100,000 PYAR
		(95%CI)		(95%CI)		(95%CI)		(95%CI)		(95%CI)		(95%CI)
All	6,532	68.30	5,657	56.86	6,599	68.77	4,482	44.90	1,861	19.46	2,491	25.07
		(66.66–69.85)		(55.39–58.37)		(67.12–70.46)		(43.59–46.24)		(18.59–20.37)		(24.10–26.08)
**Age groups**												
16–19 years	2	0.38	1	0.22	1	0.19	0	0	8	1.54	11	2.44
		(0.05–1.39)		(0.01–1.23)		(0.00–1.07)				(0.66–3.03)		(1.22–4.36)
20–29	29	2.04	20	1.43	1	0.07	6	0.43	53	3.66	130	9.32
		(1.37–2.93)		(0.87–2.21)		(0.00–0.39)		(0.16–0.82)		(2.73–4.80)		(7.79–11.07)
30–39	65	3.47	59	3.21	28	1.49	25	1.36	146	7.79	312	16.97
		(2.68–4.42)		(2.45–4.15)		(0.99–2.16)		(0.88–2.01)		(6.58–9.16)		(15.14–18.97)
40–49	243	13.64	239	13.75	189	10.60	135	7.83	232	13.02	392	22.70
		(11.98–15.46)		(12.06–15.62)		(9.15–12.23)		(6.57–9.27)		(11.40–14.81)		(20.50–25.06)
50–59	904	56.03	682	42.98	783	48.35	579	36.14	390	24.22	476	29.98
		(52.44–59.81)		(39.81–46.32)		(45.01–51.87)		(33.23–39.22)		(21.87–26.74)		(27.34–32.80)
60–69	1769	150.38	1209	99.30	1778	150.46	1109	90.02	394	33.39	432	35.59
		(143.43–157.57)		(93.77–105.07)		(143.52–157.64)		(85.63–96.44)		(30.17–36.87)		(32.31–39.11)
70–79	2270	284.41	1791	179.82	2551	317.76	1642	163.65	409	50.98	420	42.05
		(272.83–296.35)		(171.59–188.35)		(305.53–330.34)		(155.81–171.78)		(46.17–56.17)		(38.13–46.27)
80+	1250	345.87	1656	230.36	1268	347.25	986	136.26	229	63.44	318	44.20
		(326.88–365.67)		(219.35–241.78)		(328.28–367.02)		(127.84–145.09)		(55.49–72.21)		(39.44–49.33)
**Deprivation**												
Q1	1713	65.80	1428	53.13	1291	49.26	790	29.07	644	24.68	833	31.00
		(62.72–69.00)		(50.41–55.96)		(46.60–52.03)		(27.07–31.19)		(22.81–26.66)		(28.93–33.18)
Q2	1580	73.54	1386	61.63	1307	60.70	858	38.12	504	23.54	655	29.21
		(69.95–77.27)		(58.42–64.97)		(57.44–64.09)		(35.61–40.77)		(21.53–25.68)		(27.02–31.54)
Q3	1329	67.84	1162	56.53	1377	69.93	923	44.75	352	17.93	492	24.05
		(64.24–71.60)		(53.32–59.89)		(66.27–73.74)		(41.90–47.75)		(16.10–19.91)		(21.97–26.27)
Q4	1159	68.00	1044	58.53	1527	89.57	1039	57.98	225	13.25	315	17.61
		(64.13–72.04)		(55.03–62.19)		(85.13–94.19)		(54.50–61.62)		(11.58–15.10)		(15.71–19.67)
Q5	751	65.46	637	54.33	1097	95.49	872	74.67	136	11.86	196	16.75
		(60.85–70.31)		(50.18–58.74)		(89.92–101.32)		(69.78–79.80)		(9.95–14.02)		(14.48–19.27)

The gender difference in the number of lung cancer cases recorded between 1997 and 2006 was greater than that in CRC; 60% (n = 6,599) of recorded cases were men and 40% (n = 4,482) were women. Median age at diagnosis was similar in men and women (median age: M = 72 years (IQR 64–78); F = 73 years (IQR 64–79)). The overall rate of lung cancer cases recorded in men was 68.77 (95%CI 67.12–70.46) per 100,000 person years and 44.90 (95%CI 43.59–46.24) per 100,000 person years in women. Lung cancer rates were higher in men than women in all deprivation quintiles.

In contrast to CRC and lung cancer, more women (n = 2,491, 57%) than men (n = 1,861, 43%) had a recorded diagnosis of malignant melanoma during the study period. Men were older (median age 62 years, IQR = 50–73) than women (58 years, IQR = 45–72) when first diagnosed. Between 1997 and 2006, the overall rate of malignant melanoma cases recorded was 19.46 (95% CI 18.59–20.37) per 100,000 person years in men and 25.07 (95%CI 24.10–26.08) in women. More women were diagnosed with malignant melanoma than men in younger age groups, but more men were diagnosed amongst those aged 70 years and over (Table 1). Rates were lower in men than in women across all deprivation quintiles.

### 2. Survival time following cancer diagnosis by gender

**Figure 1:**
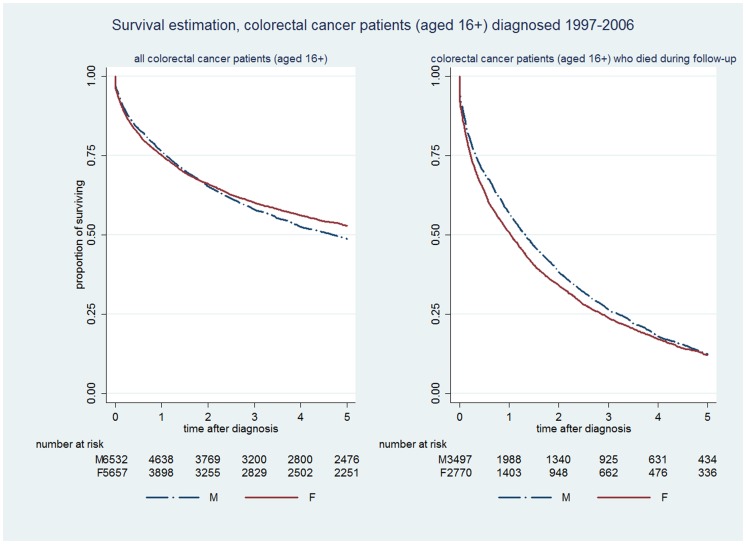
Kaplan-Meier survival curves for colorectal cancer patients (diagnosed between 1997–2006).

Figure 1 shows Kaplan-Meier curves for survival by gender, first for all those with a recorded diagnosis of CRC between 1997 and 2006, and secondly for the subset of cases who died (all cause mortality) at some time during follow up. Amongst all cases, gender differences in CRC survival to 5 years were relatively small; 49% of men and 53% of women survived at least 5 years following cancer diagnosis (Figure 1, left). Women were more likely to have survived after diagnosis (Chi-square, X^2^ = 13.12, p = 0.003); nonetheless, during the first three years following diagnosis the survival curves for men and women were very similar. Amongst the 3,497 (56%) men and 2,770 (44%) women who died at some stage during follow-up, the Kaplan-Meier curves show that a higher proportion of *women* than men died within the 1^st^, 2^nd^ and 3^rd^ year after cancer diagnosis (Figure 1, right). Within this subgroup of CRC patients, the probability of survival was better in *men* than in women (X^2^ = 6.55, p = 0.0105).

**Figure 2:**
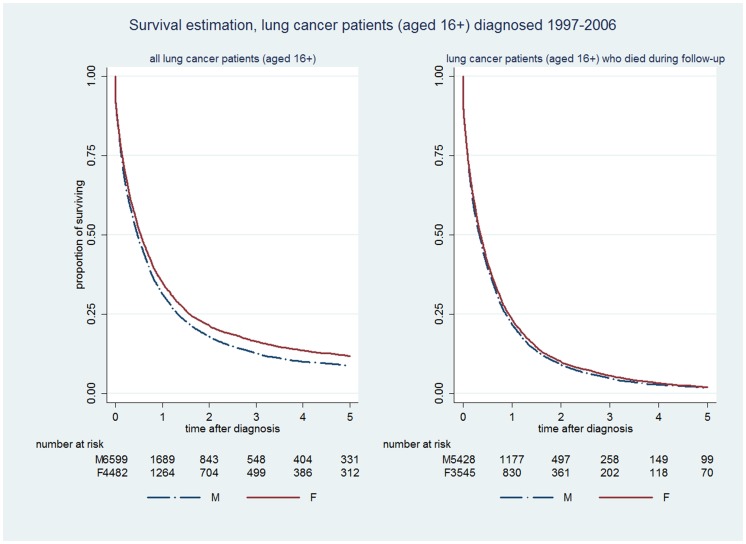
Kaplan-Meier survival curves for lung cancer patients (diagnosed between 1997–2006).

As expected, survival to five years was poorer for lung cancer patients than CRC patients: only 9% of men and 12% of women with a recorded lung cancer diagnosis survived for 5 years or more following diagnosis (Figure 2, left) and again differences in survival between men and women were relatively small(X^2^ = 24.82, p<0.001). There was negligible difference in survival to 5 years by gender amongst the subset (5,427 (60%) men and 3,544 (40%) women) who had died at some time over the whole follow-up period, (Figure 2, right) (X^2^ = 4.15, p = 0.0416).

**Figure 3:**
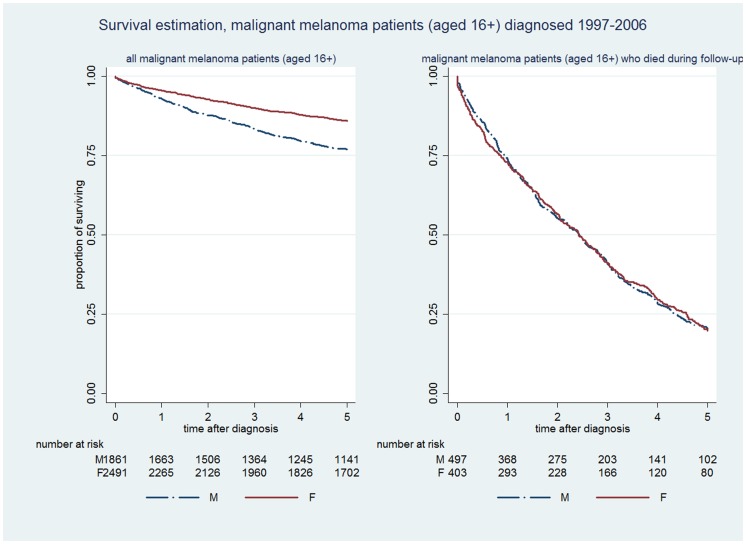
Kaplan-Meier survival curves for malignant melanoma patients (diagnosed between 1997–2006).

Five year survival rates for patients with malignant melanoma were substantially better than for CRC and lung cancer, although fewer men (77%) than women (86%) survived at least 5 years (Figure 3, left) (X^2^ = 52.58, p<0.001). However, amongst the subset of 497 (55%) men and 403 (45%) women who had died at some time over the follow-up period, there was no evidence of gender difference in time to death up to five years following diagnosis (Figure 3, right) (X^2^ = 0.01, p = 0.9360).

Table 2 shows the relative hazard of death (unadjusted, and adjusted for age and deprivation status) amongst patients with each cancer, before and after excluding patients whose dates of death and diagnosis were on the same day, and before and after excluding patients who were still alive. The adjusted male to female hazard ratio of death in all patients was 1.20, 1.24 and 1.73 for colorectal cancer, lung cancer and malignant melanoma respectively. Excluding patients whose date of diagnosis and death were recorded as the same day made little difference to the hazard ratios for CRC and lung cancer, but the hazard ratio increased for malignant melanoma. However, amongst patients who died during the follow up period, gender differences in the relative hazard of death were close to unity for lung cancer and melanoma, and 0.88 for CRC.

**Table 2:** Estimation of the relative hazard of death for patients with colorectal cancer, lung cancer and malignant melanoma, diagnosed 1997–2006.

	Crude HR (95% CI)	Adjusted HR^1,2^ (95%CI)
**Colorectal Cancer**		
All patients (M/F)	1.09 (1.02 to 1.17)	1.20 (1.12 to 1.29)
	p = 0.016	p<0.001
All patients, excluding those whose death is recorded as day of diagnosis^3^	1.12 (1.05 to 1.20)	1.21 (1.13 to 1.30)
	P = 0.001	p<0.001
Patients with fatalities (died during follow up period)	0.86 (0.80 to 0.92)	0.88 (0.83 to 0.95)
	p<0.001	p = 0.0004
**Lung Cancer**		
All patients	1.24 (1.16 to 1.33)	1.24 (1.16 to 1.33)
	p<0.001	p<0.001
All patients, excluding those whose death is recorded as day of diagnosis^4^	1.23 (1.15 to 1.31)	1.22 (1.15 to 1.30)
	p<0.001	p<0.001
Patients with fatalities (died in during follow up period)	1.07 (1.01 to 1.14)	1.08 (1.01 to 1.15)
	p = 0.0271	p = 0.0207
**Malignant Melanoma**		
All patients	1.89 (1.64 to 2.18)	1.73 (1.51 to 1.99)
	p<0.001	p<0.001
All patients, excluding those whose death is recorded as day of diagnosis^5^	2.19 (1.81 to 2.65)	1.93 (1.62 to 2.31)
	p<0.001	p<0.001
Patients with fatalities (died in during follow up period)	1.04 (0.90 to 1.19)	1.04 (0.91 to 1.20)
	p = 0.5999	p = 0.5325

1. adjusted for age (as continuous variable) and socioeconomic status.

2. including a generalized random intercept term for each patient to account for observed overdispersion.

3. 210 (3.2%) of men and 210 (3.71%) of women, p = 0.14.

4. 553 (8.4%) of men and 367 (8.2%) of women, p = 0.73.

5. 11 (0.6%) of men and 13(0.5%) of women, p = 0.84.

In summary, these analyses provide little evidence that men are being diagnosed at a later stage, since the five year survival curves do not differ greatly by gender (either in the complete patient series or amongst the subset of patients who had died during follow-up).

### 3. Primary care consultation preceding cancer diagnosis

Within the 24 months preceding cancer diagnosis, 11,787 out of all 12,189 CRC patients diagnosed between 1997 and 2006 made a total of 127,862 primary care consultations; 10,744 of all 11,081 lung cancer patients made a total 139,625 consultations; and 4,216 out of 4,352 malignant melanoma patients made a total of 37,687 consultations. Table 3 shows, for each of the three cancers, the number and percentage of men and women who had consulted in the periods 1–6 months, 7–12 months, 13–18 months, 19–24 months and 1–24 months prior to their recorded diagnosis. The analysis was carried out first amongst all cancer cases diagnosed between 1997 and 2006, and then for the subsample of cancer patients who died at some time following diagnosis. The consultation patterns were broadly similar in these two groups of cancer patients, and therefore we present here the pattern observed in *all* cancer patients. Results on consultation pattern amongst the subset of cancer patients who died during follow up are available on request.

**Table 3:** Number and percentage of cancer patients (aged 16+) who consulted during specified time periods preceding cancer diagnosis in primary care by gender.

	Colorectal Cancer			Lung Cancer			Malignant melanoma		
	No. of patients consulted (%)		Chi-square	No. of patients consulted (%)		Chi-square	No. of patients consulted (%)		Chi-square
Time prior to cancer diagnosis	M (n = 6,532)	F (n = 5,657)		M (n = 6,599)	F (n = 4,482)		M (n = 1,861)	F (n = 2,491)	
1–6 months	6138 (93.97)	5367 (94.87)	4.69(p = 0.030)	6298(95.44)	4262(95.09)	0.72(p = 0.397)	1745(93.77)	2350(94.34)	0.63(p = 0.428)
7–12	4101(62.78)	3832(67.74)	32.76(p<0.001)	4338 (65.74)	3082 (68.76)	11.05(p = 0.001)	1039(55.83)	1594(63.99)	29.68(p<0.001)
13–18	3708(56.77)	3420(60.46)	16.99(p<0.001)	3879 (58.78)	2799 (62.45)	15.00(p<0.001)	967(51.96)	1422(57.09)	11.30(p = 0.001)
19–24	3347(51.24)	3064(54.16)	10.39(p = 0.001)	3515 (53.27)	2564 (57.21)	16.74(p<0.001)	860(46.21)	1292(51.87)	13.63(p<0.001)
1–24 months	6301(96.46)	5486(97.00)	2.51(p = 0.113)	6418 (97.25)	4326 (96.52)	4.93(p = 0.026)	1803(96.93)	2413(96.87)	0.0008 (p = 0.978)

For each cancer, there is little difference in the percentage of men and women who consulted in the first six months prior to their recorded cancer diagnosis, or in the 24 months prior to diagnosis. However, a higher percentage of women than men consulted in each of the six month time periods between 7 and 24 months prior to diagnosis, although these differences were often relatively small (Table 3).

Table 4 shows the mean number of consultations made over the same time periods prior to diagnosis. In the 24 months prior to diagnosis, the mean number of consultations was a little lower for men than women. Hence, for CRC it was 9.97 (95%CI 9.89–10.04) in men and 11.09 (95% CI 11.00–11.18) in women; for lung cancer it was 12.08 (95%CI 12.00–12.17) for men and 13.36 (95% CI 13.25–13.47) for women; and for malignant melanoma it was 8.22 (95%CI 8.09–8.36) for men and 8.98 (95%CI 8.87–9.10) for women. Moreover, there was little gender differences in mean number of consultations was seen across each of the time periods (maximum M:F ratio 0.99; minimum M:F ratio 0.90, table 4). Table 5 presents the mean number of consultations in each calendar month prior to cancer diagnosis by gender and cancer site. In both men and women, the highest mean number of consultations was in the month prior to the first record of the cancer diagnosis.

**Table 4:** Mean number of primary care consultations per cancer patient (aged 16+) had within specified time periods preceding their recorded cancer diagnosis by gender.

	Colorectal Cancer			Lung Cancer			Malignant melanoma		
	Mean no. of consultation/per cancer patient consulted(95%CI)		Crude M:F rate ratio (95%CI)	Mean No. of consultation/per cancer patient consulted(95%CI)		Crude M:F rate ratio (95%CI)	Mean No. of consultation/per cancer patient consulted(95%CI)		Crude M:F rate ratio (95%CI)
Time prior to cancer diagnosis	M	F		M	F		M	F	
1–6 months	4.45(4.40–4.50)	4.69(4.64–4.75)	0.95(0.93–0.96)	5.72(5.66–5.78)	6.01(5.93–6.08)	0.95(0.94–0.97)	3.68(3.59–3.77)	3.78(3.70–3.86)	0.97(0.94–1.00)
7–12	3.31(3.25–3.36)	3.52(3.46–3.58)	0.94(0.92–0.96)	3.64(3.58–3.69)	3.91(3.84–3.98)	0.93 0.91–0.95)	2.95(2.85–3.05)	2.98(2.90–3.06)	0.99(0.95–1.03)
13–18	3.04(2.99–3.10)	3.40(3.34–3.58)	0.90(0.87–0.92)	3.45(3.39–3.51)	3.69(3.63–3.76)	0.93 (0.91–0.96)	2.84(2.74–2.95)	2.89(2.81–2.98)	0.98(0.94–1.03)
19–24	2.91(2.85–2.97)	3.24(3.17–3.30)	0.90(0.87–0.92)	3.31(3.25–3.36)	3.59(3.51–3.66)	0.92(0.90–0.95)	2.75(2.64–2.86)	2.83(2.74–2.92)	0.97(0.93–1.02)
1–24 months	9.97(9.89–10.04)	11.09(11.00–11.18)	0.90(0.89–0.91)	12.08(12.00–12.17)	13.36(13.25–13.47)	0.90 (0.89–0.91)	8.22(8.09–8.36)	8.98(8.87–9.10)	0.92(0.90–0.93)

**Table 5:** Mean number of primary care consultations per cancer patient (aged 16+) had in each month preceding their recorded cancer diagnosis by gender.

	Colorectal Cancer		Lung Cancer		Malignant melanoma	
	Mean no. of consultation/per cancer patient consulted(95%CI)		Mean no. of consultation/per cancer patient consulted(95%CI)		Mean no. of consultation/per cancer patient consulted(95%CI)	
Time prior to cancer diagnosis	M	F	M	F	M	F
1 month	1.78 (1.75–1.82)	1.81 (1.77–1.85)	2.18 (2.14–2.22)	2.21 (2.16–2.25)	1.76 (1.69–1.83)	1.73 (1.67–1.79)
2	1.60 (1.56–1.64)	1.61 (1.56–1.65)	1.89 (1.85–1.94)	1.89 (1.84–1.94)	1.41 (1.33–1.49)	1.43 (1.36–1.50)
3	1.55 (1.51–1.60)	1.58 (1.54–1.63)	1.71 (1.67–1.76)	1.74 (1.68–1.79)	1.41 (1.32–1.51)	1.36 (1.29–1.44)
4	1.41 (1.37–1.46)	1.49 (1.44–1.54)	1.59 (1.54–1.64)	1.58 (1.53–1.64)	1.37 (1.27–1.47)	1.32 (1.25–1.41)
5	1.37 (1.33–1.42)	1.41 (1.36–1.46)	1.49 (1.44–1.54)	1.51 (1.46–1.57)	1.32 (1.23–1.43)	1.30 (1.22–1.39)
6	1.35 (1.30–1.40)	1.41 (1.35–1.46)	1.45 (1.41–1.50)	1.45 (1.40–1.51)	1.29 (1.18–1.40)	1.30 (1.22–1.39)
7	1.31 (1.26–1.36)	1.36 (1.31–1.42)	1.40 (1.35–1.45)	1.46 (1.40–1.52)	1.22 (1.12–1.33)	1.26 (1.18–1.35)
8	1.33 (1.28–1.38)	1.37 (1.32–1.43)	1.39 (1.34–1.45)	1.44 (1.39–1.50)	1.29 (1.19–1.40)	1.31 (1.23–1.40)
9	1.32 (1.27–1.37)	1.37 (1.32–1.43)	1.37 (1.32–1.43)	1.42 (1.36–1.49)	1.25 (1.15–1.35)	1.23 (1.15–1.32)
10	1.26 (1.21–1.31)	1.37 (1.32–1.43)	1.40 (1.34–1.45)	1.39 (1.33–1.45)	1.27 (1.17–1.38)	1.24 (1.15–1.32)
11	1.25 (1.20–1.31)	1.33 (1.28–1.39)	1.38 (1.33–1.44)	1.29 (1.23–1.35)	1.23 (1.13–1.34)	1.21 (1.12–1.29)
12	1.28 (1.23–1.34)	1.32 (1.27–1.38)	1.37 (1.31–1.42)	1.34 (1.29–1.41)	1.17 (1.06–1.27)	1.20 (1.12–1.29)
13	1.26 (1.21–1.31)	1.31 (1.25–1.36)	1.38 (1.33–1.44)	1.32 (1.26–1.38)	1.25 (1.14–1.36)	1.21 (1.13–1.30)
14	1.21 (1.16–1.27)	1.29 (1.24–1.35)	1.31 (1.26–1.37)	1.29 (1.23–1.35)	1.25 (1.15–1.37)	1.17 (1.08–1.26)
15	1.24 (1.19–1.30)	1.35 (1.29–1.41)	1.35 (1.29–1.40)	1.36 (1.30–1.42)	1.24 (1.14–1.35)	1.20 (1.11–1.29)
16	1.22 (1.16–1.28)	1.33 (1.28–1.39)	1.32 (1.26–1.37)	1.32 (1.25–1.38)	1.15 (1.04–1.26)	1.24 (1.16–1.33)
17	1.21 (1.16–1.27)	1.30 (1.24–1.36)	1.30 (1.24–1.35)	1.34 (1.28–1.41)	1.18 (1.07–1.30)	1.26 (1.16–1.35)
18	1.23 (1.18–1.29)	1.33 (1.27–1.39)	1.33 (1.28–1.39)	1.32 (1.26–1.39)	1.18 (1.07–1.29)	1.22 (1.13–1.30)
19	1.20 (1.15–1.26)	1.30 (1.25–1.36)	1.28 (1.23–1.34)	1.27 (1.21–1.34)	1.22 (1.11–1.33)	1.19 (1.10–1.28)
20	1.20 (1.15–1.26)	1.29 (1.23–1.35)	1.32 (1.26–1.38)	1.37 (1.30–1.43)	1.20 (1.09–1.31)	1.26 (1.17–1.36)
21	1.21 (1.15–1.26)	1.28 (1.22–1.34)	1.30 (1.25–1.36)	1.30 (1.24–1.37)	1.22 (1.10–1.34)	1.16 (1.07–1.26)
22	1.18 (1.12–1.23)	1.26 (1.20–1.32)	1.32 (1.26–1.38)	1.33 (1.27–1.40)	1.19 (1.08–1.31)	1.23 (1.13–1.32)
23	1.16 (1.10–1.22)	1.25 (1.19–1.32)	1.29 (1.23–1.35)	1.35 (1.28–1.41)	1.08 (0.97–1.20)	1.16 (1.07–1.26)
24	1.17 (1.11–1.23)	1.21 (1.16–1.28)	1.29 (1.24–1.35)	1.30 (1.24–1.37)	1.11 (1.01–1.23)	1.19 (1.10–1.29)

**Figure 4:**
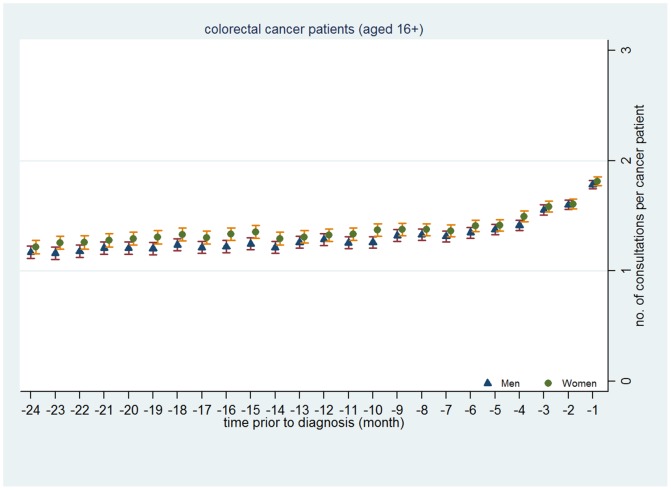
Mean number of primary care consultations per colorectal cancer patient made prior to cancer diagnosis by month and gender.

**Figure 5:**
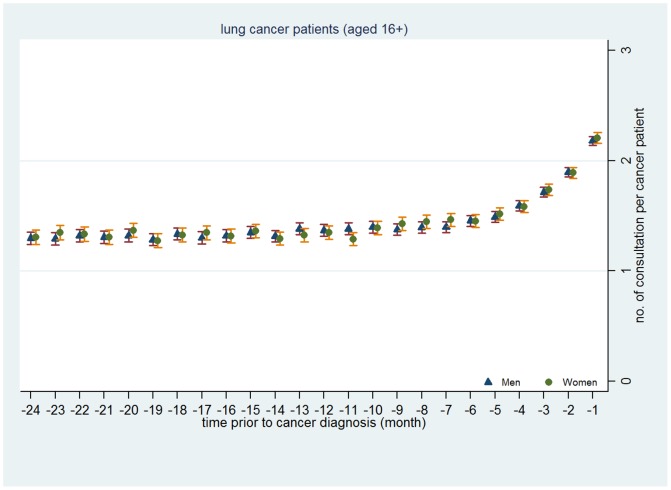
Mean number of primary care consultations per lung cancer patient made prior to cancer diagnosis by month and gender.

**Figure 6:**
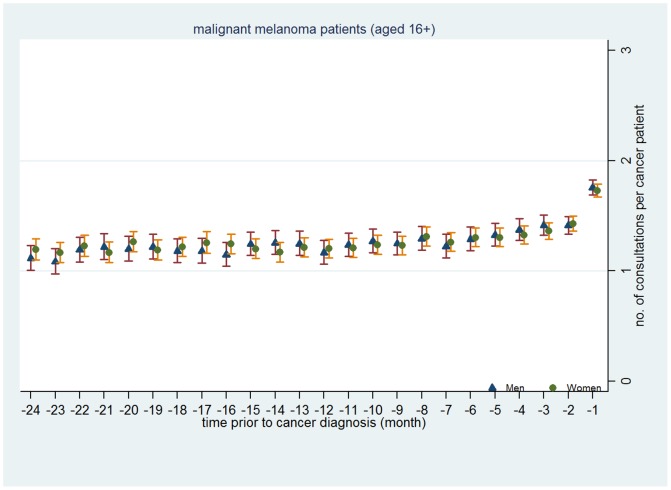
Mean number of primary care consultations per malignant melanoma patient made prior to cancer diagnosis by month and gender.

Figures 4 to 6 presents mean number of consultations by gender each month in the 24 months preceding diagnosis for CRC (Figure 4), lung cancer (Figure 5) and malignant melanoma (Figure 6) graphically. These clearly illustrate the lack of gender difference in consulting prior to diagnosis.

In summary, these analyses suggest that whilst men consult a little less than women prior to a diagnosis with CRC, lung cancer or malignant melanoma, these differences are surprisingly modest.

## Discussion

Our aim in this study was to assess whether there is evidence to support the hypothesis that gender differences in patterns and timing of consulting for symptoms of three non-sex specific cancers (colorectal, lung and malignant melanoma) could plausibly account for gender differences in mortality. We investigated whether there were gender differences in survival following diagnosis with these three cancers, and examined in particular time to death in the first few years after diagnosis. We then examined the number of GP contacts within the 24 months preceding diagnosis by gender. Our analyses provide little evidence that men are being diagnosed at a later stage than women with these three cancers, since the five year survival curves do not differ greatly by gender (either in the complete patient series or amongst the subset of patients who had died during follow-up). We have also shown that whilst men perhaps consult a little less than women in the time periods 7–12, 13–18 and 19–24 months prior to a diagnosis with CRC, lung cancer or malignant melanoma, these differences are surprisingly modest. In summary, we contend that our analyses of these three non-sex specific cancers provide little support for the hypothesis that gender differences in mortality might be explained by men presenting later or less often to primary care.

As expected, we found that more men than women were diagnosed with colorectal cancer and lung cancer, but more women with malignant melanoma (at least at younger ages). Our results confirm some male disadvantage in relation to cancer, as reported by others using different data sources [16], [28], [29]. There was a small overall cancer survival advantage of females, which remained when the analyses were adjusted for age and socio-economic status. Amongst colorectal cancer patients, male to female hazard of death increased slightly from 1.09 to 1.20 when adjustment was made for age and social deprivation, perhaps because women tend to develop colorectal cancer at an older age compared to men [30]–[33].

Some studies have suggested that adjustment for stage of cancer attenuates gender differences [34]–[37]. Since stage of cancer is not reliably recorded in the THIN database, we identified a subgroup of cancer patients who subsequently died after diagnosis, as a proxy for patients with similar severity. Amongst these patients, there was no evidence that men were dying earlier within the first 5 years after diagnosis, as we would have expected if they were presenting, and being diagnosed, at a later stage of disease. For CRC patients, the direction of gender disparities in the hazard of death reversed from 1.20 amongst all CRC patients to 0.88 in the subgroup who had subsequently died. Our finding that, for colorectal cancer men in this subgroup were in fact better survivors than women parallels findings from a German study which reported better survival from advanced colorectal cancer in men than in women, but better survival in patients with localised cancers in women than men [36]. Likewise, a study of colorectal cancer in Europe has reported a female to male relative risk of death three years from diagnosis of 1.01 when adjusted for age, gender, cancer site, stage and determinants of stage [38]. Variations in cancer survival may of course be explained both by stage at diagnosis and quality of care following diagnosis.

It is still commonly asserted that men's under-use of health services and their tendency to ‘under-report’ health problems put them at greater risk of dying from cancers [39], although there is greater recognition that this is unlikely to be the case given the lack of evidence to support the contention [40]. Another important finding of this study is that differences in rates of consulting prior to cancer diagnosis were negligible between men and women diagnosed with these three non sex-specific cancers. This mirrors results from our earlier study which noted that gender differences in the use of health services reduce considerably (to less than 10%), when comparing men and women with similar underlying morbidity [8]. We would argue that the marginal gender differences in consulting shown in the current study strongly challenge the hypothesis that gender differences in primary care utilisation are an important explanation for gender differences in longevity; they provide little support for an effect of gender on the promptness of consulting [41]. Nonetheless, individuals' pathways prior to a cancer diagnosis are often not straightforward, and patients vary in timing of their visits to medical professionals [42]. Both the number of consultations prior to cancer diagnosis and the time from first symptom onset to first presentation to a GP have been used in recent analyses as a measure of promptness of cancer diagnosis [43], [44], although these measures present different challenges both for measurement and interpretation [44]. Keeble and colleagues demonstrate that there is considerable variation by cancer type in promptness of presentation amongst people diagnosed with 18 types of cancers using data from the National Audit of Cancer Diagnosis in Primary Care in England in 2009–2010. They analyse ‘patient interval’ data (i.e. “the period between first symptom onset and first relevant presentation to a doctor”) rather than ‘primary care interval’ data (i.e. “the promptness with which general practitioners suspect the diagnosis of cancer and refer patients to specialists”) [44]. Prompt presentation was most frequent for bladder and renal cancer and least frequent for oropharyngeal and oesophageal cancer. However, their multivariable analysis showed *no* evidence for variation in promptness of presentation by gender and no evidence of interaction between cancer diagnosis and gender. Another paper examining variation in the number of GP consultations prior to a hospital referral for cancer did find gender differences. This study utilised data from the 2010 National Patient Cancer Experience Survey in England to investigate which factors explained the considerable variation in the number of GP consultations before hospital referral for 24 types of cancer. They reported that the probability of making three or more pre-referral consultations was greater for women than men (OR 1.28, 95% CI 1.21–1.36; p<0.001), and this was seen for most of the 18 cancers occurring in both men and women with a “few notable exceptions”. The effect was particularly marked for bladder cancer (OR 2.31, 95% CI 1.98–2.69). These findings thus suggest that the “readiness of general practitioners to suspect cancer” not only varies by cancer type, but also by gender; for some cancers, particularly bladder cancer where there appears to be a particular “danger of misattributing urinary tract symptoms in women to a benign cause” (p363), women may ‘need’ to make more visits to their GP before cancer is considered as the underlying cause for reported symptoms [45]. These findings, together with our own, suggest the need for further careful investigation of gender differences in the responses of both patients and health care professionals to the experience, reporting and attribution of symptoms which could be indicative of an underlying malignancy.

The strengths of our study include the use of a UK wide primary care database and population-based electronic medical records of cancer cases in general practice, the recording of which are comparable to national cancer registry data [26], [27]. Nonetheless, the study has several potential limitations. First, there is the lack of reliable information on cancer stage at diagnosis, as this is rarely recorded on primary care systems. To address this limitation we conducted our survival analyses both in all cancer patients and in the subsample who had died during follow-up. In the latter group there was no evidence of poorer male survival in the first few years following diagnosis, whilst we would have expected to see a difference if men were being diagnosed at a later stage of disease. Ideally, we would examine stage of disease directly. Second, although age and deprivation were adjusted for in cancer survival analyses, we were not able to examine to what extent gender differences in cancer survival vary by factors such as treatment and co-morbidities. Studies have found significant links between co-morbidities and cancer outcomes amongst these three cancers [46]–[48], and cancer survival may be influenced by gender differences in co-morbidity at cancer diagnosis [49]–[52]. Third, our relative hazard estimates of death were based on all cause deaths rather than cancer specific deaths, as the recording of cause of death is often incomplete in GP records [53]. Nevertheless, THIN recording allows us to identify all deaths amongst people diagnosed with these three cancers, irrespective of whether they are directly or indirectly related to the cancer, not subject to bias, providing a robust estimate of relative gender differences in cancer survival. Finally, our analyses used GP recorded data on consultations, but the content and nature of individual consultations is unknown. Although some of these consultations may have been for other symptoms, arguably the patient's attendance at the doctor should present an opportunity for the doctor to detect symptoms of cancer, irrespective of gender.

## Conclusions

This large population-based study confirmed a relatively modest disadvantage amongst men in five year survival from three non-sex specific cancers compared to women in the UK. However, amongst the subset of cancer patients who died during follow-up there was very little difference in survival by gender. Furthermore, patterns of consulting prior to cancer diagnosis differed little for men and women, suggesting that gender differences in survival are unlikely to be explained by gender differences in consultation for these cancers at least. These findings thus challenge the still common assumption that men delay seeking care for serious illness and are being diagnosed at a later stage of disease leads to a poorer prognosis and hence their higher overall mortality and reduced life expectancy. Further research is needed to confirm whether this holds for other types of cancer, and to understand the discrepancy in cancer survival between men and women in order to prepare the way for appropriate interventions to eradicate gender inequalities.
